# Eco-Friendly and Sustainable Pathways to Photoluminescent Carbon Quantum Dots (CQDs)

**DOI:** 10.3390/nano13030554

**Published:** 2023-01-30

**Authors:** Shikha Gulati, Arikta Baul, Anoushka Amar, Rachit Wadhwa, Sanjay Kumar, Rajender S. Varma

**Affiliations:** 1 Department of Chemistry, Sri Venkateswara College, University of Delhi, Delhi 110021, India; 2 Institute for Nanomaterials, Advanced Technologies, and Innovation (CxI), Technical University of Liberec (TUL), Studentská 1402/2, 461 17 Liberec, Czech Republic

**Keywords:** carbon quantum dots (CQDs), photoluminescent, bottom-up approach, top-down approach, green synthesis

## Abstract

Carbon quantum dots (CQDs), a new family of photoluminescent 0D NPs, have recently received a lot of attention. They have enormous future potential due to their unique properties, which include low toxicity, high conductivity, and biocompatibility and accordingly can be used as a feasible replacement for conventional materials deployed in various optoelectronic, biomedical, and energy applications. The most recent trends and advancements in the synthesizing and setup of photoluminescent CQDs using environmentally friendly methods are thoroughly discussed in this review. The eco-friendly synthetic processes are emphasized, with a focus on biomass-derived precursors. Modification possibilities for creating newer physicochemical properties among different CQDs are also presented, along with a brief conceptual overview. The extensive amount of writings on them found in the literature explains their exceptional competence in a variety of fields, making these nanomaterials promising alternatives for real-world applications. Furthermore, the benefits, drawbacks, and opportunities for CQDs are discussed, with an emphasis on their future prospects in this emerging research field.

## 1. Introduction

Carbon-based nanomaterials have gained tremendous global attention from researchers, owing to their unique properties, namely high conductivity, low toxicity, biocompatibility, and easy functionalization [1,2,3,4,5,6,7,8]. This consideration can be attributed to their harmless effect on the environment when compared to other commonly deployed energy sources emanating from fossil fuels [9,10,11,12,13]. Since the discovery of fullerenes in the 1980s, material science and engineering related to carbon materials have piqued the public’s interest. Thus, these carbon-based substances, such as graphene quantum dots (GQDs), carbon nanodots, polymer dots, and other carbon-derived dots, are explored widely for their promising applications in carbon fixation, gas storage, adsorbents, and other fields [14,15]. Perhaps, out of this carbon family, the most commonly reported member is GQDs, which are made out of graphene, with lateral dimensions less than 100 nm, and possess some common properties of graphene and C-dots [16]. To this point, GQDs are often confused with carbon dots (C-dots), but they possess certain differences; GQD framework consists of sp^2^-hybridized carbon, whereas C-dots possess sp^3^-hybridized carbon [17]. Furthermore, C-dots are quasi-spherical carbon nanoparticles that have graphene and turbostratic carbon mixed in different ratios [18]. Realizing the potential of GQDs, researchers across the globe have suggested a plethora of techniques for the preparation of GQDs, but many of them involve harsh and non-environmentally friendly approaches, thus necessitating the search for eco-friendly methods. Over a few decades, industrial chemical research has turned its focus towards the use of renewable sources and waste-reduction approaches, leading to the evolvement of a new concept, termed green chemistry, which, endowed with its 12 principles, directs the chemical industry along eco-sustainability pathways. One of the significant goals, as stated in the seventh principle, entails the design of synthetic approaches with low environmental impact, without the use of harmful reagents or solvents. On this account, renewable feedstocks, or abundant and biodegradable raw materials that are commonly accessible from natural sources represent the appropriate direction for the greener production of CQDs, exploiting the usage of alternate activation energy sources, such as microwave and ultrasound irradiation; this contrasts the unsustainable production from non-renewable fossil resources, such as oil, coal, and natural gas [19]. In view of our resolute interest in the field of nanomaterials and green chemistry [20,21,22,23,24,25,26,27,28,29], this review focuses on eco-friendly synthesis methods for assorted CQDs and uncovering their promising applications. Different fabrication methods comprise molecular oxidation, intermolecular coupling, and the use of microwave or ultrasonic irradiation, among others, with a special emphasis on biomass-derived precursors [17,30,31,32,33]. These methods have their own merits and demerits that require special attention depending on the need of a particular application. Furthermore, modification strategies are discussed through different pathways to develop novel physicochemical properties among different CQDs, with brief coverage of their structural analysis. The various means of exploiting carbon precursors to generate CQDs are discussed in detail, along with the evaluation of their future outlook. Adding to the existing literature [34,35,36], this review will serve as a resource for methodologies to synthesize novel carbonaceous materials, particularly GQDs, following the precepts of green chemistry. There are several review papers on the title of carbon quantum dots, but our paper collectively highlights the recent synthetic advancements in the creation of the graphene quantum dots during the past 10 years (2012–2021); a compilation of the previous 7 years of research in this field, in a very readable and an easy-to-understand format, is emphasized. Additionally, Table 1, Table 2, Table 3, Table 4, Table 5, Table 6, Table 7, Table 8 and Table 9 provide a consolidated form of assorted research papers, segregated by synthesis options for GQDs, thus making it easy for the readers to acquire the gist of prior work that is cited here.

## 2. Graphene Quantum Dots (GQDs): An Incomparable Nanomaterial

**Figure 1:**
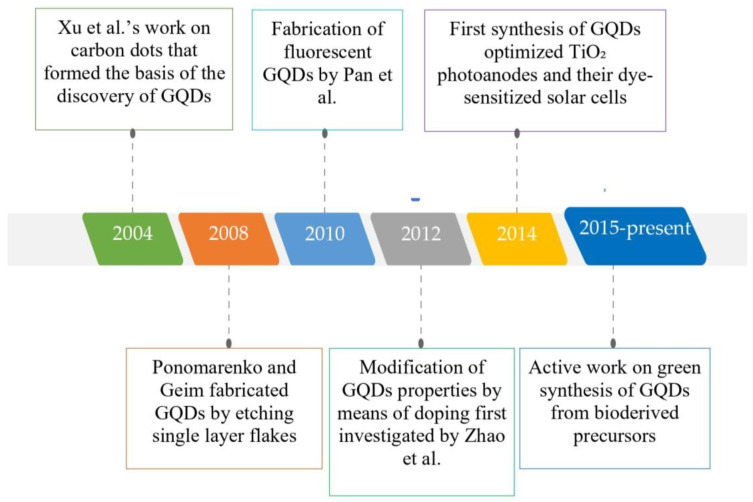
A glimpse of the progress of graphene quantum dots. The timeline is as follows: (i) Xu et al.’s work on carbon dots that formed the basis for the discovery of GQDs (2004) [45]; (ii) Ponomarenko and Geim prepared GQDs by etching single-layer flakes (2008) [37]; (iii) Pan et al. first fabricated fluorescent GQDs (2010) [16]; (iv) Dong et al. developed photoluminescence (PL) GQDs by tuning the carbonization degree of citric acid, popular research forming the basis for the green synthesis of GQDs (2012) [46]; (v) first synthesis of GQDs optimized TiO_2_ photoanodes and their dye-sensitized solar cells (2014) [47]; and (vi) active work on green synthesis of GQDs from bioderived renewable materials (2015–present).

Graphene quantum dots (GQDs), first synthesized by Ponomarenko and Geim in 2008 [37], are zero-dimensional nanomaterials that are characterized by an atomically thin graphitic plane and have exceptional luminescence properties. They are small fragments of graphene with a two-dimensional (2D) lateral size (<100 nm) [38,39]. The typical drawbacks of graphene, such as poor solubility, zero bandgaps, and a tendency to aggregate into graphite [40], can be circumvented by using GQDs, which have some favorable attributes, namely low toxicity, cost-effectiveness, advanced electrical/optical properties, etc. [41,42,43,44]. Since their discovery, GQDs have made tremendous progress, as illustrated in Figure 1.

**Figure 2:**
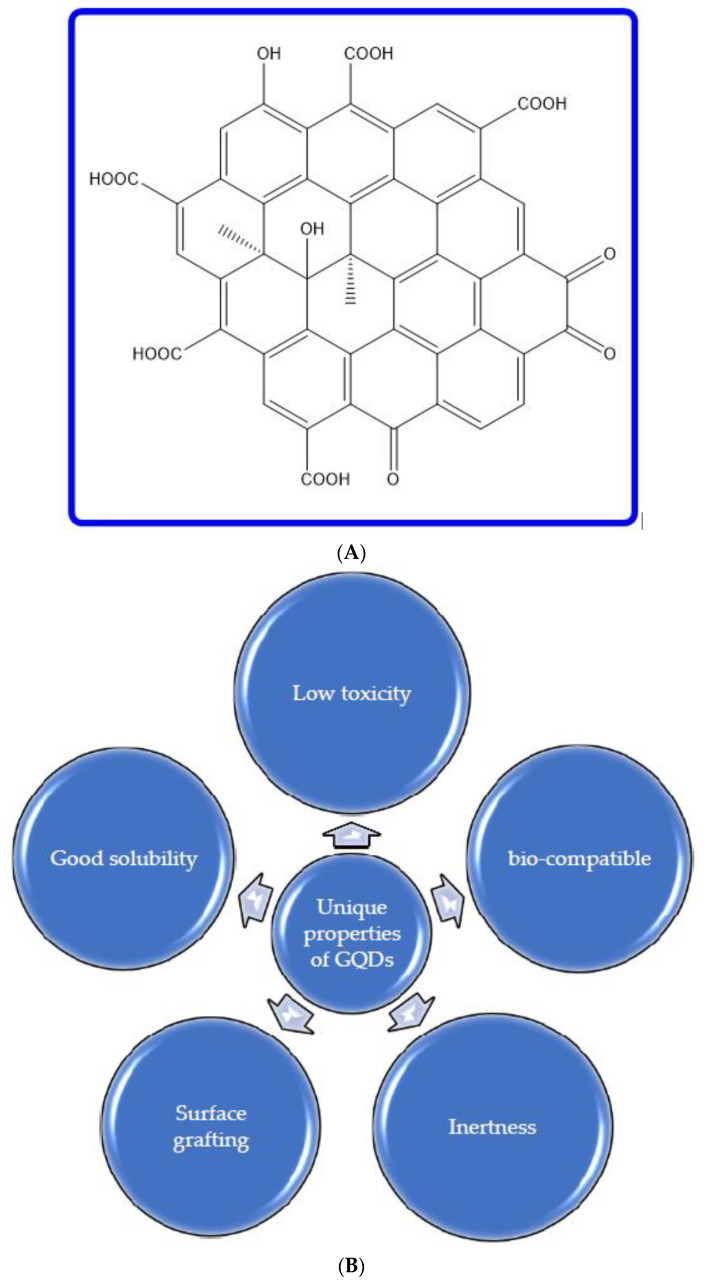
(**A**) Structure of GQDs. (**B**) Unique properties of GQDs.

GQDs are formed by converting two-dimensional graphene to a zero-dimensional form through a number of processes. Due to the zero dimensionality of GQDs, they exhibit very good quantum confinement and edge effects, with the most important attribute being a non-zero bandgap material, which has been one of the major drawbacks of graphene. Thus, GQDs have all the properties of graphene, with a non-zero bandgap, which renders them very useful for numerous significant appliances, including photovoltaics, biological imaging, and others. The bandgap of GQDs can be modulated by varying the doping process and changing the dopant, thus providing a wide array of options for obtaining GQDs. Figure 2 depicts the structure of GQDs and summarizes the favorable properties of GQDs that make them exceptional candidates for numerous appliances in biomedicine [25,48], energy [12,49], and optoelectronic devices [38,50].

## 3. Environmentally Friendly Synthesis of GQDs: Greener and Sustainable Approaches

GQDs have been fabricated by employing numerous synthesis approaches, broadly divided into two categories—top-down and bottom-up. However, the traditional synthetic methods demanding chemical precursors, concentrated alkali/acid treatments, toxic organic solvents, etc., can cause major harm to the environment. This calls for the need to synthesize quantum dots in a sustainable manner, so that hazardous chemical precursors and organic solvents, which cause environmental pollution, can be avoided. Quantum dots are ideally synthesized in a sustainable way since this strategy offers several advantages, such as the use of low-cost, naturally available raw materials with lower toxicity, simple operations, and uncomplicated postprocessing. The deployment of renewable sources makes it possible to synthesize quantum dots without depleting Earth’s resources, thus contributing to a more sustainable and secure future. In this scenario, natural biomaterial sources are frequently favored over other inorganic, organic, or synthetic reserves in the search for an inexpensive, efficient, and environmentally friendly method of synthesizing carbon quantum dots since they are biocompatible and renewable. Consequently, over the years, the synthesis of GQDs has pivoted to the adaptation of green chemistry tenets for attaining sustainability, with the main focus being the design of products that reduce the use of toxic chemicals and hazardous chemical synthesis [1,51]; green chemistry has found its relevance in a myriad of applications, namely in the treatment of water pollution [52], pharmaceutical industry [53], synthesis routes to other nanomaterials [54], etc. Hence, the global effort has shifted its focus to fabricating GQDs (and QDs) via greener routes and deploying eco-friendly bioderived precursors [26,27,55,56].

### 3.1. Strategic Selection of Greener Bioderived Precursors for Eco-Friendly Synthesis of GQDs

One of the most crucial steps in the process of synthesizing GQDs is the selection of precursor material, as this specifically involves the use of greener precursors via carbonization or controllable methods of synthesis [57,58]. Carbon-based precursors, such as graphite, graphene, graphene oxide, fullerene, carbon fibers, etc., are derived from non-renewable sources, which include petroleum coke and coal [59,60,61], thus provoking a need to develop alternatives from renewable precursors, which include biomass waste, discarded agricultural residues, municipal waste, and food waste, for the efficient and low-cost preparation of high quality and abundant GQDs [62]. For example, some of the successful areas of growth for carbon-based materials produced from biomass waste are hydrogen storage, sorption material, biomedicine, etc. [63,64]. Graphene oxide is a commonly used precursor for its conversion into GQDs via oxidative cleavage methods assisted with microwave or ultrasonic irradiation. A significant role is played by the functional groups on graphite in breaking down C-C bonds, so only a few layers of GO sheets are obtained [16]. Some other examples of green precursors that were considered are plant extracts, agricultural residues, fruit wastes, etc.

**Figure 3:**
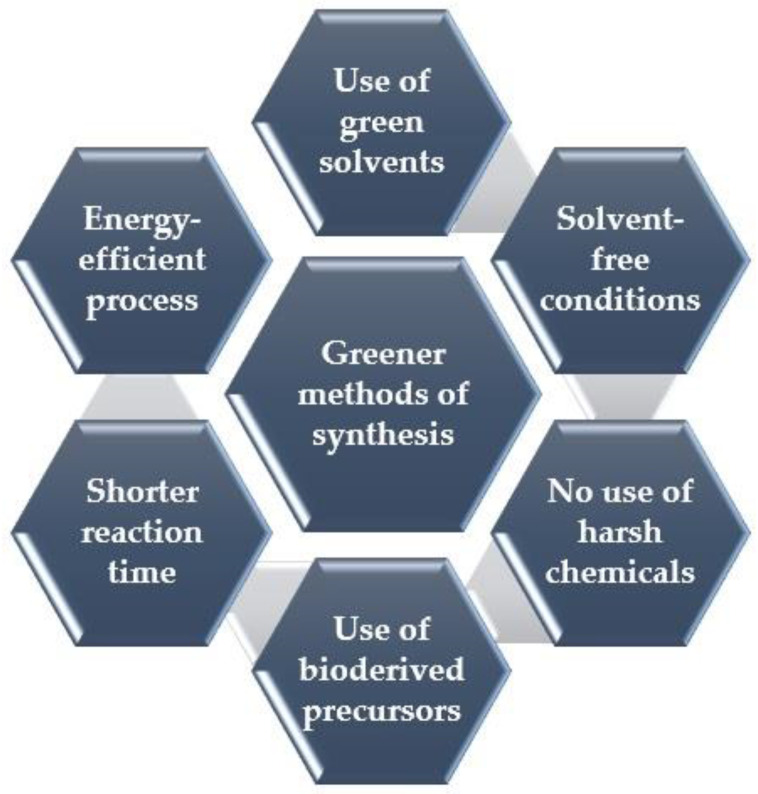
Salient attributes of a “greener” synthesis method.

Herein, numerous methods under top-down and bottom-up approaches encompassing greener aspects, such as the usage of solvent-free conditions, renewable feedstocks, microwave and ultrasonic irradiation, catalysis, etc., are highlighted in the green chemistry domain (Figure 3).

### 3.2. Diverse Routes for the Greener Synthesis of GQDs

Synthesis techniques can be broadly divided into two major groups: top-down and bottom-up. In bottom-up synthesis, sp^2^ carbon domains are obtained from organic molecules via intermolecular coupling, soft template, and microwave (MW)-assisted methods, resulting in the formation of products with a controllable size and morphology. In contrast, the main reaction in the top-down methods is the oxidation process either through the free radical or oxidative cleavage method [16], in which large sp^2^ carbon domains are converted into smaller ones, usually resulting in high-yield access to GQDs via simple, easy-to-operate, and time-saving protocols [59].

#### 3.2.1. Top-Down Approach for Preparation of GQDs

**Figure 4:**
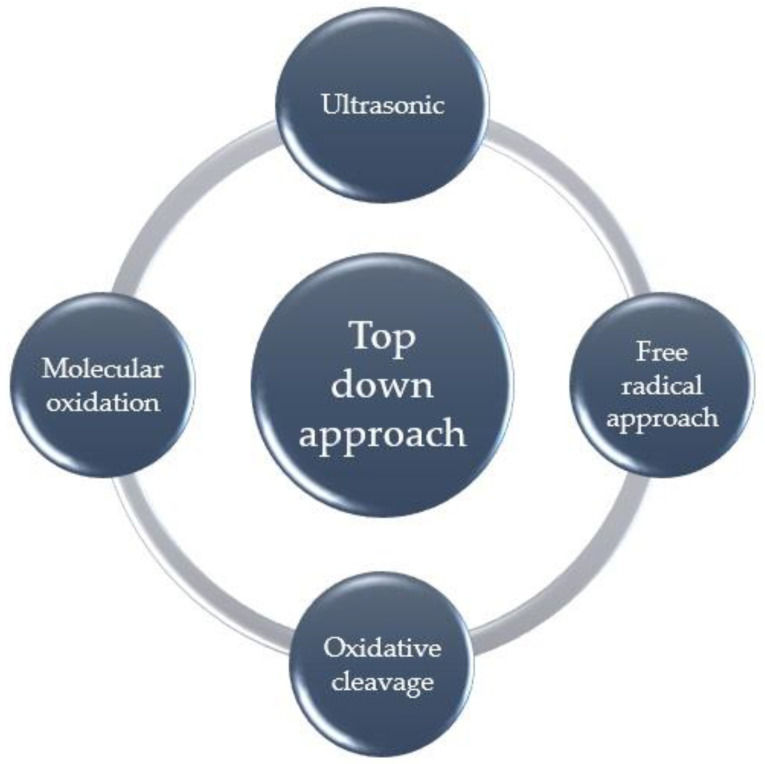
Schematic depicting the different techniques of the top-down approach used for the preparation of GQDs.

The top-down approach involves the conversion of large sp^2^ carbon domains into smaller segments, and, hence, the methods falling under this category are ultrasonic-assisted, molecular-oxidation, free-radical-oxidation-approach, and oxidative-cleavage methods (Figure 4) [65,66,67]. Different top-down approaches are discussed in this section.

##### Ultrasound-Assisted Synthesis

**Figure 5:**
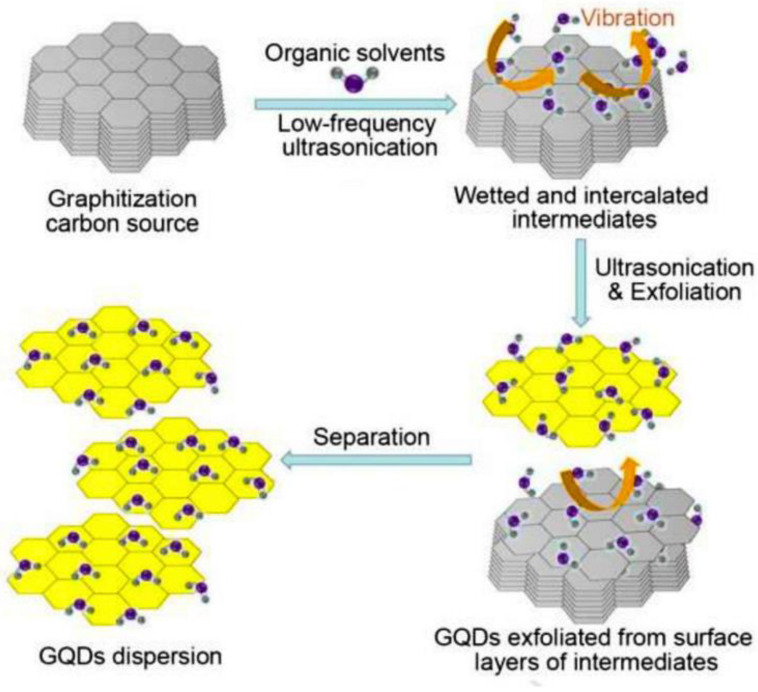
Schematic diagram showing the ultrasonic-assisted preparative process for synthesizing GQDs by using carbon precursors, such as nano-graphite and acetylene black, in organic solvents. Reprinted from Ref. [77], Copyright (2016), with permission from Elsevier.

Ultrasonic techniques for the synthesis of GQDs involve the use of intense ultrasound under drastically high temperature and pressure conditions in order to avoid a long reaction time for designing nanocarbon particles. In many studies, ultrasonic-assisted methods have been incorporated and purely deployed the use of the mechanical forces on graphene derivatives to cut them down to GQDs. The use of eco-friendly ultrasound for the synthesis/post-synthetic modification of carbon nanomaterials influences factors such as size, shape, surface structure, chemical composition, solubility, and aggregation in the process [68,69]. Zhou et al. [70] successfully prepared GQDs via a simple ultrasonic process, in the size range of 3–5 nm, exhibiting excellent PL properties, for usage as biosensors, bioimaging, laser, and light-emitting diodes. Similarly, Zhu et al. [71] successfully introduced a high-yielding synthesis of GQDs by using a simple one-step, fast, and acid-free ultrasonication method. Liquid-phase exfoliation assisted by high-energy ultrasounds was introduced as a greener option by Sarkar et al. [72]. Ultrasonic synthesis hybridized with a Sn@CQDss@Sn anode was introduced, illustrating a promising biophotonic application [68]. An innovative and efficient synthesis of -COOH-enriched GQDs based upon integrated tailoring in GO, using the O_3_/H_2_O_2_/ultrasound process, was achieved; GQDs of the size 4–10 nm were obtained, exhibiting decent PL properties, as a promising candidate in the advancement of fundamental research of QDs and applications in solar cells [73]. An ultrasonic-assisted hydrothermal strategy for the preparation of GQDs by using leather as a precursor was introduced recently for the preparation of GQDs exhibiting strong luminescent properties without doping [74]. In another approach, GQDs were prepared by grinding graphene flakes, followed by exposure to ultrasonication, with promising application in optoelectronic devices [75]. Gao et al. offered another low-cost, time-saving, and greener synthesis of three types of GQDs (PGQDs, EGQDs, and GOGQDs) by using ultrasound-assisted exfoliation methods from different forms of graphite as precursors [76]. Figure 5 depicts an expeditious and industrially relevant greener method for the preparation of defect-selective GQDs (LD-GQDs and HD-GQDs), using the ultrasonic-assisted liquid exfoliation method from graphitic carbon precursors [77].

**Figure 6:**
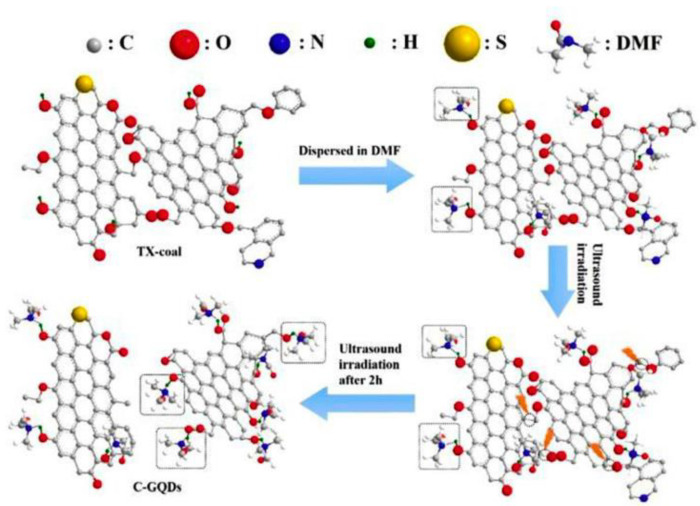
Schematic illustrating the generation of C-GQDs from coal precursors. Reprinted with permission from Ref. [79], Copyright (2019) American Chemical Society. The TaiXi anthracite coal (TXcoal) precursor was homogeneously dispersed in *N*,*N*-dimethylformamide (DMF) to rapidly form hydrogen bonds between DMF and the oxygen-containing groups of the coal molecules. Following that, the suspension was ultrasonicated in an ultrasonic cell crusher, and acoustic cavitation caused high pressure (in excess of 500 bar), intense local heating (5000 °C), and cooling rates greater than 1010 K s^−1^.

Ultrasonic assistance has been deployed in the synthesis of amino-functionalized GQDs prepared for optoelectronic applications [78]. Using anthracite coal as a precursor, Zhang et al. synthesized low-cost ultrasonic-tailored PL C-GQDs with good selectivity and sensitivity with blue emission; two PL emission peaks at 429 and 469 nm were exhibited by the C-GQDs, as illustrated in Figure 6 [79].

##### Molecular Oxidation Method for the Synthesis of GQDs

Molecular oxidation is a multifaceted method for the synthesis of GQDs in high yields wherein oxidants such as HNO_3_ tear down the sp^2^ arrangement of graphene into sp^3^ carbon domains that are further oxidized to form GQDs [80,81,82]. It has been reported that Vulcan XC-72 carbon black serves as a potential precursor which, upon refluxing with nitric acid, affords GQDs with 75% yield with a purity of 99.96 wt.% [83]. Another group reported graphene oxide (GO) as the precursor and H_2_O_2_ as an oxidant to obtain GQD products without any additional purification steps. Crude biomass is a well-known precursor for the synthesis of GQDs [32,84] as illustrated by Pan et al. via a hydrothermal route where biomass-derived carbon material is made to react with oxidizing agents that introduce epoxy groups on basal planes. From this, GQDs are obtained via a hydrothermal route that is followed by deoxidization in a basic medium [16].

Rice husks that are activated with strong acids have been subjected to hydrothermal treatment to produce GQDs with a considerable yield [32], as shown by Tian et al., using H_2_O_2_ under an *N*,*N*-dimethylformamide (DMF) environment via a solvothermal route that is devoid of concentrated acids [85]. Recently, nitrogen-doped graphene quantum dots (NGQDs) have been synthesized in a process where graphite is used as a precursor, and this reduced the overall cost of the preparation. Furthermore, ammonium persulfate is deployed as an oxidant and nitrogen source, and H_2_O_2_ as an oxidative agent in *N*-methyl-2-pyrrolidone as a solvent. The unprecedented use of solvent extraction is illustrated to purify NGQDs that displayed green and blue fluorescence; the product yield of NGQDs was ~52%, containing 88% of green-emissive NGQDs and 12% of blue-emissive NGQDs [86].

##### Free-Radical Oxidation for the Synthesis of GQDs

**Figure 7:**
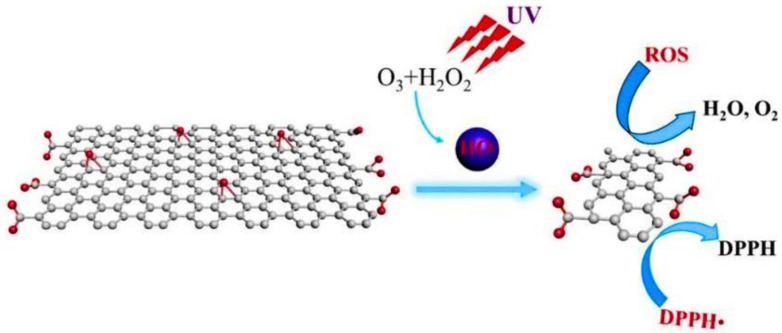
Schematic representation for the synthesis of GQDs via the free-radical method, a top-down approach. Reprinted from Ref. [97], Copyright (2020), with permission from Elsevier.

The free-radical oxidation method for the synthesis of GQDs is a clean and efficient pathway without the formation of any by-product (Figure 7); molecular oxidation and acidic intercalation destroy the organized sp^2^ structure of the natural graphite, resulting in sp^3^ carbon that is preferentially oxidized. The morphological trimming of graphene into GQDs occurs via free-radical oxidation by activities of the OH * radical that accelerate the oxidation and cutting of the sp^2^ carbon structure into a smaller domain [65,66,67,87]. The anionic oxidation of water generates OH * and O * free radicals that function as electrochemical scissors to generate GQDs [88]. Electrochemical scissoring of wood charcoal [89], carbon nanotubes [90], reduced graphene oxide (RGO) films [91], graphite rods [92], and 3D graphene [90,91,92,93] is known for its ability to produce GQDs in high yields [94].

Nirala et al. suggested an electrochemical oxidative route from biomass-derived wood charcoal to prepare GQDs; the *O_2_ and *OH free radicals that are generated cut the graphene sheets of charcoal into very small particles, termed E-GQDs [89]. Recently, tryptophan-conjugated graphene quantum dots (Trp-GQDs) were fabricated by using the oxidation protocol under greener conditions wherein the by-products produced, i.e., O_2_ and H_2_O, can be utilized for advanced biomedical applications; H_2_O_2_ radicals played an important role as a precursor for this facile synthesis method [95].

In brief, during the formation of GQDs-H, H_2_O_2_ gets dissociated to create HO_2_· radicals through anodic oxidation, and, subsequently, HO· radicals are produced at the cathode under a low electric field, as shown below:

(1) $$H_{2}O_{2}\;-\;e^{-}\;\to HO_{2}\;+\;H^{+}$$

(2) $$H_{2}O_{2}\;-\;e^{-}\;\to {\mathrm{OH}}^{-}\;+\;\mathrm{OH}$$

Ensuing HO_2_· and HO· free radicals are strong oxidizers, and they instantly oxidize C–C bonds to corresponding C-OH bonds, followed by the generation of C-O-C groups from the adjacent C-OH bonds by losing a H_2_O molecule. Properly aligned C-O-C groups on the surface of the graphite rod electrode render the graphene backbone fragile so that it could be readily attacked. The regions encompassing graphene pieces with surrounded epoxy lines and/or edges in the working electrode may further be cut smaller and smaller during CV deoxidization by the removal of O atoms from an epoxy group. Consequently, in addition to the continuous decomposition caused by H_2_O_2_, the formation of abundant C-O-C groups contributed to both accelerating electrode exfoliation and the generation of GQDs-H with ultra-small sizes [96].

##### Oxidative Cleavage Method

**Figure 8:**
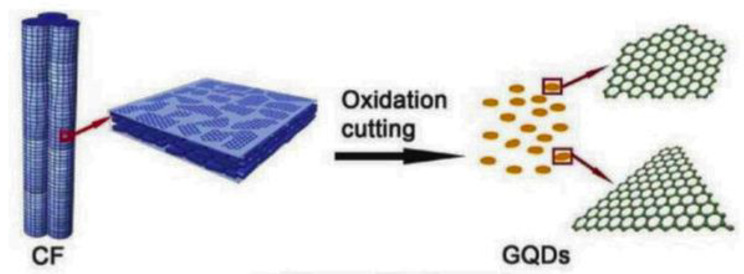
Schematic representation for the synthesis of GQDs by oxidative cutting, a top-down approach. Reprinted with permission from Ref. [18], Copyright (2012) American Chemical Society.

Oxidative cleavage (also known as oxidative cutting) is the protocol that deploys mild oxidizers to break the carbon–carbon bonds of GO, graphene, or carbon nanotubes (CNTs). A greener oxidative cleavage method involves the utilization of mild oxidizers that prove to be an eco-friendly alternative to toxic oxidants. Peng et al. [18] obtained GQDs with fluorescent properties by oxidative cleavage of carbon fiber under a high temperature of about 120 °C, which displayed a narrow distribution between 1 and 4 nm. (Figure 8).

**Figure 9:**
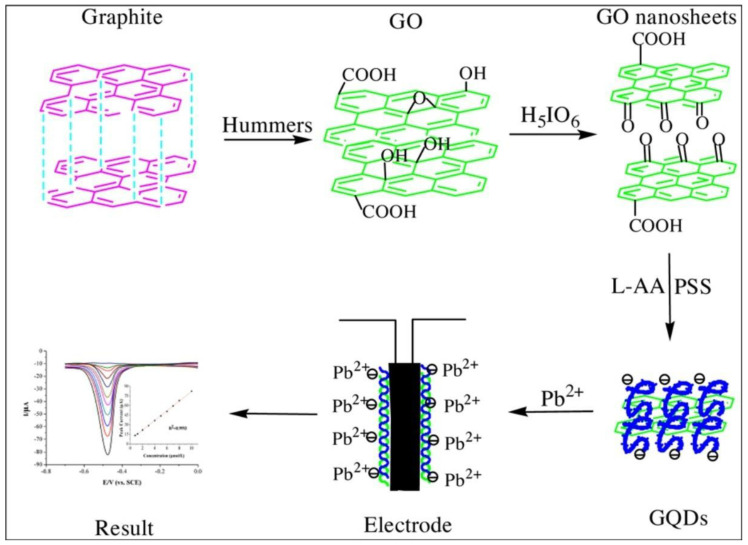
Schematic representation for the synthesis of GQDs by oxidative cleavage, a top-down approach. Reprinted from Ref. [98], Copyright (2014), with permission from Elsevier.

In an eco-friendly method, GO powder was shortened and then reduced by using sodium polystyrene sulfonate as the dispersant and L-ascorbic acid as a reducing agent, as graphically represented in Figure 9 [98].

(3) $$Graphite\;\overset{Hummers\;method}{\to }\;GO\;\overset{H^{5}IO^{6}}{\to }\;GO\;nanosheets\overset{L-ascorbic\;acid}{\underset{polystyrene\;sulfonate}{\to }}\;GQDs$$

In another study, multiwalled carbon nanotubes (MWCNTs) were made to undergo acid treatment and chemical exfoliation to generate GQDs with a uniform size distribution, 2D morphology, and zigzag edge structure [99]. Their results indicated that, upon UV excitation, the GQDs exhibited bright blue emission and also displayed good stability and high water solubility. Carbon black [100] has been used as the starting material to synthesize GQDs via an MW-assisted oxidative cleavage technique in a short time of 1 h; MW irradiation facilitated fast oxidative cleavage. GQDs with good crystallinity and with the size of ~10 nm and thickness of 2 nm were achieved and were then modified with mesoporous carbon aerogels to form a GQD/CA composite that served as a promising alternative to platinum as the counter electrode material for dye-sensitized solar cells (DSSCs).

#### 3.2.2. Bottom-Up Approach for the Preparation of GQDs

**Figure 10:**
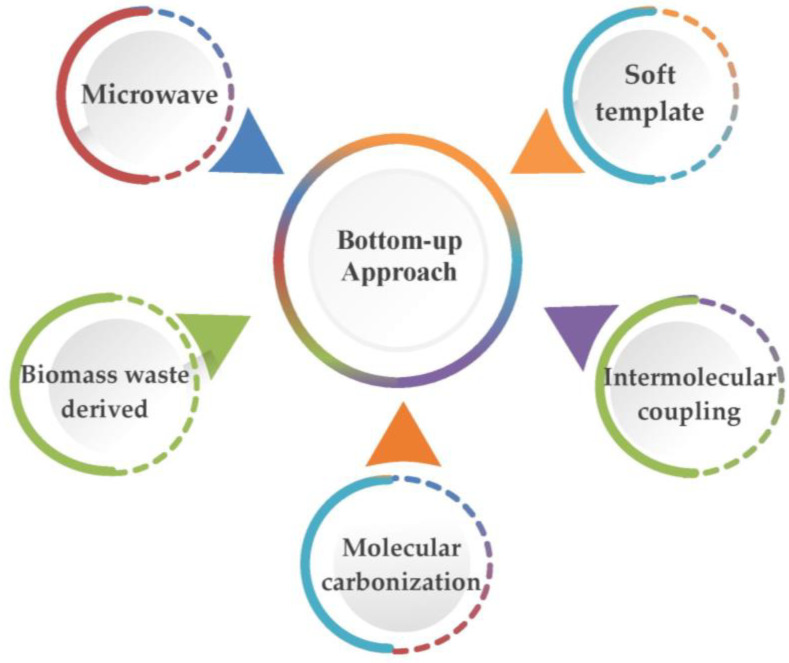
Schematic depicting the different techniques of the bottom-up approach used for the preparation of GQDs. The top-down approach involves breaking down the carbon material structures. In microwave (MW)-assisted synthesis, the frictional energy generated via molecular rotation of polar solvents by MW energy is utilized. In order to reduce the longer reaction time of hydrothermal methods, the fusion of microwaves with a hydrothermal approach, termed microwave-assisted hydrothermal (MAH) synthesis, helps in making the synthesis process swifter and more efficient. Waste/biowaste-derived sustainable synthesis of GQDs entails the carbonization of waste materials and biomass residues. Intermolecular coupling protocol has the advantage of generating GQDs with controlled size and shape. Molecular carbonization is a facile environmentally friendly technique where organic molecules are dehydrated and further carbonized.

The bottom-up approach involves the synthesis of nanomaterials by assembling basic units into larger structures and, hence, generally comprises MW-assisted, MW-assisted hydrothermal, waste-derived, intermolecular-coupling, soft-template, and molecular-carbonization protocols (Figure 10), which entail the chemical assembly of small organic molecules.

##### Microwave-Assisted Synthesis of GQDs

In microwave-assisted synthesis, the frictional energy generated via molecular rotation of polar solvents by MW energy is utilized which improves the production yield to offer uniformly sized materials in a much shorter time, thus making it an ideal option for the preparation of nanomaterials. A shorter reaction time due to the strong absorption of MW irradiation by one component of the reaction has encompassed this method of synthesis within the concept of green chemistry; added advantages are the synergy with green solvents and solvent-free conditions [101]. N-GQDs with of 5.3 nm size and displaying fluorescence (FL) peak at 390 nm have been prepared via an environmentally friendly, ultra-fast ammonia-driven MW-assisted route deploying glucose and ammonia at room temperature and atmospheric pressure [102]. Tak et al. [103] reported a one-pot MW-assisted method for the preparation of GQDs of 10 nm in size from the flowers of *Clitoria ternatea* for the treatment of Alzheimer’s disease. Li et al. [104] prepared electrochemiluminescent two-color GQDs via a MW-assisted method. gGQDs were prepared via cleavage of GO under acidic conditions with a quantum yield as high as 11.7%. Furthermore, bGQDs were synthesized via a further reduction of gGQDs with NaBH_4_ with a quantum yield (QY) up to 22.9%. An eco-friendly MW irradiation route was adopted [105] for the preparation of GQDs from low-cost organic precursors, which could then be potentially applied for the environmental remediation of triazine. Gu et al. [106] synthesized *N*-doped GQDs via a solid phase MW-assisted (SPMA) method, using urea and citric acid as precursors; a QY of 38.7% was achieved. In another work [107], an acid-free MW approach was used for synthesizing fluorescent boron-doped GQDs, using graphene oxide and borax as the carbon and boron sources, respectively; the ensued GQDs gave a fluorescent QY of 21.1% and showed bright blue photoluminescence at 330 nm.

(4) $$GO+{Na}_{2}B_{4}O_{7}\;\overset{Microwave\;irradiation}{\underset{230\;{}^{\circ}C,\;30\;\min}{\to }}\;B-GQDs$$

**Figure 11:**
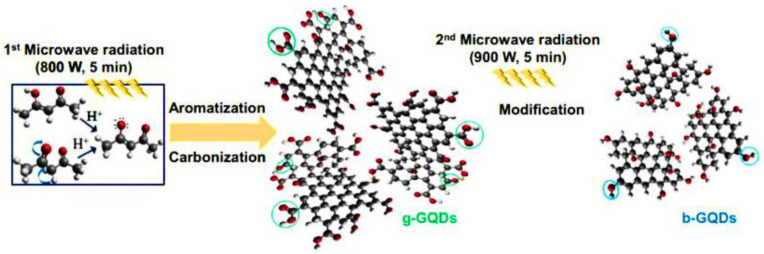
Schematic illustration for the MW-assisted synthesis of g-GQDs and b-GQDs. Reprinted from Ref. [116], Copyright (2015), with permission from Elsevier.

The MW-assisted route has been described for the synthesis of GQDs by using *Mangifera indica* (mango leaves) as the carbon precursor, the size of which ranged from 2 to 8 nm [108]. Boron-doped GQDs [109] have been prepared by using an MW-assisted method from boron carbide crystals that could potentially be used as a photoanode for dye-sensitized solar cells (DSSCs). Nguyen et al. [110] performed MW-assisted synthesis of GQDs and N-GQDs from citric acid and urea. The use of microwave irradiation provided considerable improvement in the production methods suggested earlier by Dong [46] and Qu [111], enabling the ultra-fast fabrication of the GQDs. N-doped GQDs [112] were synthesized via microwave heating by using triethanolamine and sodium citrate as the precursors. The obtained GQDs had an average diameter of 5.6 nm and gave a quantum yield of 8%. The N-GQDs showing considerable bright blue fluorescence were applied as probes for metal ion detection. Kumawat et al. [113] reported a microwave-assisted green synthesis method for the preparation of GQDs, using grape seed extract as the precursor. Their “self-assembled” GQDs could potentially be utilized for cell proliferation and photoluminescent sensing nucleus imaging. GQDs were prepared by Shin et al. [114] via microwave irradiation, using graphite as the precursor. In a study [115], ultra-rapid microwave-assisted methods were reported for the preparation of bright fluorescent GQDs within three minutes. The MA-GQDs exhibited a quantum yield of up to 35% which have potential applications in live-cell staining and white LEDs. As illustrated in Figure 11, Umrao et al. [116] reported an MW route for the preparation of green GQDs and blue luminescent GQDs, using acetylacetone, an organic solvent, as the precursor, thus eluding the drawbacks of most of the bottom-up approaches. Zhuang and co-workers [117] converted solid citric acid to GQDs under MW-irradiation conditions; the as-prepared GQDs were successfully utilized for live-cell imaging.

##### MW-Assisted Hydrothermal Method for the Synthesis of GQDs

**Figure 12:**
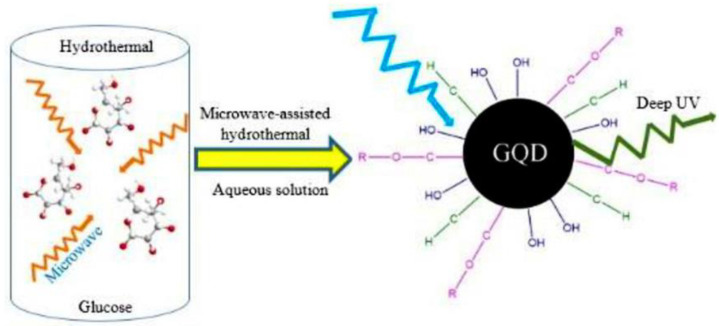
Illustration of synthesis of GQDs by MW-assisted hydrothermal technique. Adapted with permission from Ref. [120], Copyright (2012) American Chemical Society. A simple MW-assisted hydrothermal method was used to create glucose-derived water-soluble crystalline graphene quantum dots (GQDs) with an average diameter of 1.65 nm (5 layers).

In order to reduce the longer reaction time of hydrothermal methods, the fusion of microwaves with a hydrothermal approach, termed microwave-assisted hydrothermal (MAH) synthesis, helps in making the synthesis swifter and more efficient wherein expeditious and homogenous heating by microwaves leads to the swift formation of quantum dots of uniform size, thus realizing the beneficial attributes of both hydrothermal and MW approaches [118,119]. Lau’s group [120] (Figure 12) prepared glucose-derived water-soluble GQDs. They showed the largest emission energy (E = 4.1 eV) among other QDs which were synthesized via the MAH approach, using glucose as the precursor; the average diameter of the synthesized GQDs was 1.65 nm, and they had a PL quantum yield of 7–11% [120]. A similar study on GQDs deployed a soft-template MAH method to study size dependence on the structure and optical properties and the realization of the highest QY of 15% [121]. Using a two-step MAH approach, Chen et al. [122] reported the first pyrrole-ring-surface-functionalized GQDs (p-GQDs). Sun et al. used this approach, and fluorinated GO was used as the raw material to synthesize both the fluorinated and nonfluorinated GQDs [123]. GQDs were synthesized by using the MAH route. The approach involved MW heating of citric acid for 20 min, followed by ultrasonic treatment for 2 h [124]. Using the same approach, Hou et al. prepared oxygen-rich N-doped GQDs, using glucose and urea as precursors; the PL QY of 5.2% was observed [125]. For the application of electrochemical dopamine sensing, Ben Aoun et al. prepared N-doped GQDs with the basic MAH method, using glucose as a precursor to prepare N-doped GQDs—chitosan nanocomposites [126]. In another work, white fluorescent GQDs were prepared by a facile two-step MAH approach, using graphite as a precursor for its application in white-light-emitting diodes (WLEDs) [127]. The same synthesis method was utilized in another work [128] where the as-prepared GQDs were modified with adenine for application in efficient two-photon bioimaging. Gómez et al. synthesized GQDs following a fast microwave-assisted hydrothermal protocol, using glucose and ammonium hydroxide [129].

##### Biomass-Waste-Derived Method of the Synthesis of GQDs

Waste/biowaste-derived sustainable synthesis of GQDs involves the carbonization of waste materials and biomass residues as carbon sources obtainable from a number of waste resources. Conversion of these precursors to GQDs follows a bottom-up route. Some of the bio-precursors which have been used for the synthesis of different kinds of GQDs/CQDs comprise watermelon peels, orange juice, dairy waste, fruit waste, sweet pepper, expired drugs, etc.; this is an ideal strategy for the preparation of GQDs due to their sustainability, renewability, abundance, and low-cost which add to their green attributes [31]. Mishra et al. managed to synthesize carbon dots by using bike pollutant soot [130] wherein UV–Visible spectroscopy, photoluminescence spectroscopy, TEM, UV–Visible light chamber, and FTIR studies were deployed to characterize the synthesized carbon quantum dots; novelty lies in electrical humidity sensor proposed by using the waste pollutant soot. Rajamanikandan et al. synthesized blue emissive CQDs from biowaste peels [131]. CQDs are synthesized from agro-waste *Ananas comosus* using a simple hydrothermal treatment in this study and powder X-ray diffraction, Fourier-transform infrared, UV–Visible spectral analysis, and quantum yield measurements are utilized to characterize the synthesized CQDs. Singh et al. synthesized CQDs from lemon-peel waste, using a simple and low-cost hydrothermal process [132]; they are endowed with excellent photoluminescence properties, and a high aqueous stability, with a quantum yield (QY) of around 14%. Ye, Xiang, et al. used the most abundant and affordable energy resource to synthesize GQDs, using a low-cost facile one-step synthesis; three types of coal were used, anthracite (a), bituminous coal (b), and coke (c) [59]. Similarly, Kang et al. prepared GOQDs through a facile one-step green pulsed laser ablation in liquid (PLAL) process, using low-cost coal as the carbon source for bioimaging applications, a method devoid of using strongly acidic/basic solutions; excellent photostability, biocompatibility, optoelectronic properties, and low toxicity were revealed [133]. Roy et al. used a plant-leaf-based one-pot hydrothermal method to prepare GQDs for their application in white LEDs; Neem and Fenugreek were the two green plant leaves that were used, and the ensued GQDs exhibited a high QY of 41.2%, and 38.9%, respectively, and displayed an intense green and pH-independent PL [31]. Another study was conducted on a relatively large-scale synthesis of Am-GQDs (Amine terminated GQDs) by pyrolysis of biowaste, followed by its hydrothermal treatment [33]. Inspired by this work, Zhang et al. prepared *N*-GQDs for sensing and bioimaging applications, using marigold as the precursor, by drying marigold granules in an oven, at 60 °C, followed by the pyrolysis of the powder obtained [134]. The latest research also exploited marigolds to synthesize GQDs, refraining from the use of any hazardous chemical [135]. As-synthesized GQDs showed excellent prospects in supercapacitors, and these are elaborated upon later in the article (*Tagetes erecta*). A demonstration of a relatively mild preparation of 2D GOQDs from a renewable and cheap precursor, i.e., a waste paper, was provided by Adolfsson et al. [136]. CTP (coal tar pitch) is a by-product obtained in the coking industries that was exploited by Liu, Zhang, et al. for developing GQDs via their mild oxidation using hydrogen peroxide; high yield (>80 wt.%) of highly soluble and strongly fluorescent GQDs were obtained [137]. Jlassi et al. [138] prepared N-S co-doped GQDs from graphitic waste in presence of ammonia. Mohan et al. synthesized GQDs by using expended sugarcane bagasse, which was subjected to an improvised Hummers’ Method, followed by the formation of a GQD/SnO_2_ nanocomposite via a hydrothermal route. Various surface defects were observed on the prepared GQD/SnO_2_ nanocomposite, considerably enhancing their antibacterial properties and making them a suitable candidate for potential application in the sanitation industry [139]. Ding et al. delivered the gram-scale synthesis of lignin-derived GQDs by using a two-step method in which the synthesis entails oxidation cleavage, followed by the aromatic refusion of alkali lignin particles. Lignin is considered a biomass waste that has been used here to prepare high-quality GQDs via a green, low-cost, and large-scale synthesis protocol for deployment as multicolored fluorescent probes for bioimaging applications [140]. Chen et al. developed a greener method for GQDs comprising cellulose polymer and water, without the use of any chemicals. High PL emission, hydrophilicity, and low cytotoxicity of the synthesized GQDs made it a favorite for application in bioimaging and biolabeling [69]. Rahul et al. synthesized N-doped GQDs derived from waste precursors, melamine sponge, and arjuna bark, using microwave treatment [141].

##### Intermolecular Coupling Method

The intermolecular coupling [84,142,143,144] protocol has the advantage of allowing researchers to prepare GQDs with a controlled size and shape; small organic entities are heated above their melting point, leading to condensation and nucleation, followed by the formation of GQDs; organic salts [145,146], coffee grounds [84], citric acid [147], ascorbic acid [148], and glycerol [80], among others, serve as potential precursors for this technique. Kalita et al. reported the synthesis of GQDs of 2–6.5 nm in size from rice grains in a controlled manner [149]. The ensued oligomers, upon thermal degradation, successively grow at high temperatures to form GQDs; growth is controlled by the heat treatment time that further helps in the tunable size distribution of GQDs. The preparation of tunable-sized GQDs has been reported via the emulsion template method, using honey carbohydrates as the starting material [150]. Single-layer graphene quantum dots (SLGQDs) were prepared with DI water as the solvent and glucose as the precursor [151]. At first, dehydration of glucose was accomplished, creating C=C and the basic unit of the graphene structure. Secondly, the interaction between the hydrogen atoms of a glucose molecule with the hydroxyl group of adjacent glucose molecules occurred to form water. Lastly, the covalent interaction of carbon atoms leads to the formation of GQDs [152]. In another report, the carbonization degree of citric acid was tuned, and carbonized products were dispersed into alkaline solutions to prepare GQDs [33]. Naik et al. [149,153] synthesized GQDs via the pyrolysis of citric acid with the addition of NaOH at a later stage to maintain a suitable pH range for GQD fabrication; hydronium ion produced by acid acted as an auto-catalyst in the next decomposition reaction. The aromatization and formation of aromatic clusters occur via aldol condensation and cycloaddition and the subsequent addition of NaOH leading to GQD formation at different pHs which plays a vital role in the formation of GQDs from citric acid. Interestingly, in these bottom-up processes, the doping effects of heteroatoms can be achieved easily from precursor materials, an advantage in broadening their application to more storage devices. For example, *N*-doped GQDs have been prepared by hydrothermal techniques, using citric acid as a source of carbon and ethanolamine, diethylene amine, ethylene diamine, or urea as a source of nitrogen [111]. Likewise, sulfur-doped monodispersed GQDs have been obtained through hydrothermal treatment of fructose, using sulfuric acid as a sulfur source [154].

##### Molecular Carbonization Method

**Figure 13:**
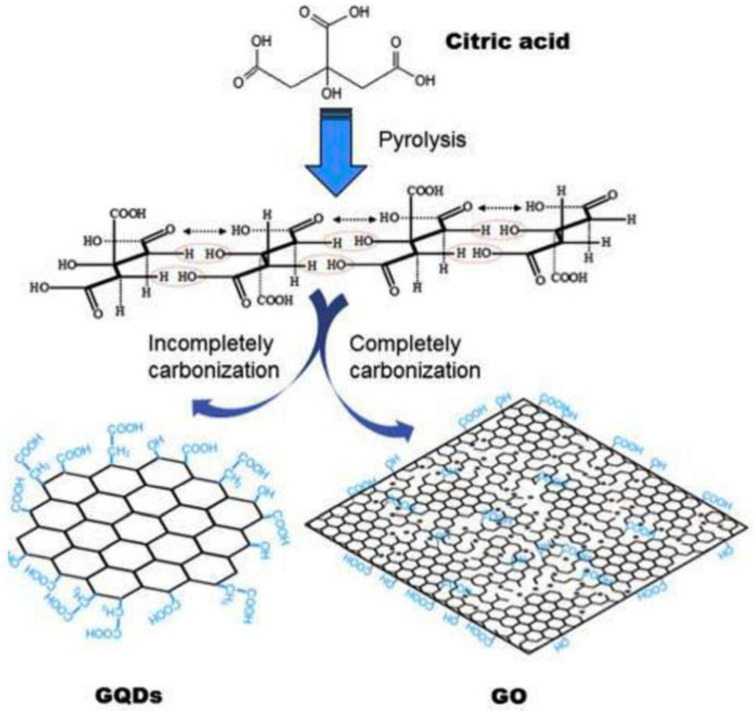
Synthesis of GQDs by carbonization of citric acid, a bottom-up approach. Reprinted from Ref. [46], Copyright (2012), with permission from Elsevier.

Molecular carbonization is a facile environmentally friendly technique in which organic molecules are dehydrated and further carbonized. The use of carbon-rich residues from organic molecules as precursors makes this method a favorable green synthesis method. As depicted in Figure 13, Dong et al. [46] reported a simple carbonization technique for the synthesis of GQDs by tuning the degree of carbonization of citric acid (CA) and then dispersing the products into alkaline solutions. Solid citric acid was first heated in a breaker at 200 °C, transitioning its color change from colorless to pale yellow and then to orange, thereby suggesting the formation of GQDs with a width of 15 nm and a thickness of 0.5 to 2 nm, that displayed a strong photoluminescence QY of 9%. The above-mentioned GQDs synthesis method was used by Taher and co-author [155], Amjadi et al. [156], Arvand et al. [157], Diao et al. [158], Jian et al. [159], Tashkhourian et al. [160], and Teymourinia et al. [161]. Teymourinia et al. [161] prepared GQDs by using a green precursor, i.e., corn powder in deionized water, which was heated in a stainless autoclave at 180 °C for 8 h; GQDs with a diameter of 20 to 30 nm were obtained after centrifugation of the solution. The same group [162] applied the as-synthesized GQDs for improving light harvesting in DSSCs by exploiting its property to display considerable luminance that could convert UV light to visible light. The solar cells based on the prepared GQDs showed greater power conversion efficiency as compared to reference cells. Graphene quantum dots were applied for Fe^3+^ detection in another work [163], wherein *N*-doped GQDs were synthesized by carbonization of CA via hydrothermal treatment in the presence of ammonia. The method proposed by Qu et al. [111] was deployed to prepare N-GQDs and applied to electrochemical sensing of anti-cancerous hydroxyurea. N-and-P-co-doped GQDs have been prepared by using ATP as a precursor by heating ATP powder for 1 h, at 900 °C, leading to its carbonization [164]. Subsequent chemical exfoliation of the carbonized ATP with HNO_3_ for 24 h, followed by centrifugation and dissolution in deionized water afforded the ATP-GQDs.

(5) $$ATP\;\underset{90{}^{\circ}C,\;1\;hour}{\overset{Carbonization}{\to }}\;Carbonized\;ATP\;\underset{{HNO}_{3},\;24\;hours}{\overset{Exfoliation}{\to }}\;GQD$$

Gu et al. [165] prepared blue-emitting *N*-GQDs of ~5–10 nm in size by tuning the carbonization degree of CA and subsequent doping of nitrogen into the graphene lattice. In the literature [152,166,167,168] precursors such as orange juice, organic pollutant 4-nitrophenol, deionized water and glucose, and asphalt, respectively, have been utilized for the fabrication of GQDs. In another investigation [169], nine types of organic solvents (ethylenediamine, glycerol, ethylene glycol, methanol, dimethyl formamide, acetone, ethanol, toluene, and carbon tetrachloride) were used as models to examine their carbonization towards GQDs. The as-prepared GQDs displayed good stability, smaller size distribution, unconverted FL, and excitation wavelength-tunable.

##### Soft Template Method

**Figure 14:**
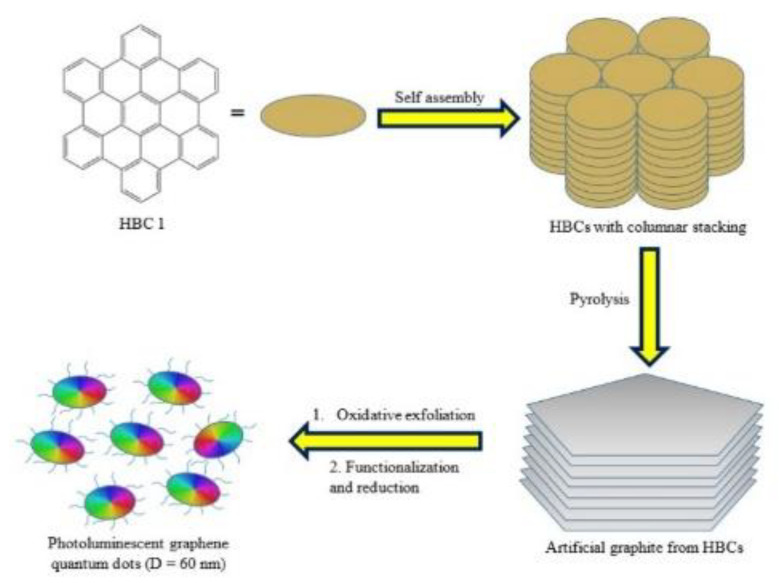
Synthesis of GQDs from HBC via soft-template route. Adapted with permission from Ref. [173], Copyright (2011), American Chemical Society.

The soft template method is a popular, low-cost, and environmentally friendly method of synthesis of GQDs that may be favorable for mass production with the key advantage of control over the morphology, structure, and size of the synthesized nanomaterials. This green method of synthesis does not require the use of any harsh chemicals and involves the use of a template as a scaffold for assisting the growth of nanostructures and finally removal of the template [170,171]. Yang and co-author [172] synthesized N-GQDs by employing the soft template method, using 1,3,5-triamino-2,4,6-trinitrobenzene (TATB) as the carbon source and template which was annealed in the thermal process, resulting in the generation of expanding gases and break down of chemical bonds. As a result of the expanding gases, multilayer TATB was developed into a single layer to produce the N-GQDs. As depicted in Figure 14, Mullen et al. [173] utilized hexa-peri-hexabenzocoronene (HBC) as the precursor to synthesize monodispersed disk-like GQDs with a uniform size of 60 nm, where during pyrolysis, HBC was condensed, forming a graphitic framework. A modified Hummer’s method was then applied for oxidizing and exfoliating the artificial graphene, followed by its reduction with hydrazine; thus, GQDs with a thickness of 2–3 nm were obtained by using HBC as a template under excitation at 365 nm, and the GQDs exhibited a strong blue PL emission. Gao et al. [174] fabricated sulfur-doped GQDs by using small molecular carbon disulfide as a template. Carbon powder was formed by burning a liquid mixture, and it was then used to exfoliate the GQDs. In another work [175], GQDs were synthesized, using citric acid as the template; as-prepared GQDs showed a high quantum yield of 83%. In another study [176], zinc-histidine-functionalized GQDs were fabricated via the thermal pyrolysis of CA and histidine. The as-synthesized GQDs displayed high water solubility, with an average size of 3.2 nm, and were applied for the adsorption of Cu^2+^. This method was deployed for synthesizing His-GQD and Zn-His-GQDs, which were then employed for the preparation of His-GQD-GMA [177]. The study opened a gateway to meet the needs of future applications, such as energy storage and conversion devices, etc.

#### 3.2.3. Acid-Free Method

As discussed in the previous methods for synthesis, some of these methods involve the use of strong acids which need to be removed post-synthesis by various complicated purification processes [70]. Herein, we throw light on some examples of top-down and bottom-up methods of the synthesis of GQDs that refrain from using any acidic chemical in the process, i.e., acid-free methods of synthesis. These methods are environmentally friendly, with easy purification and workup, in securing high yields of 0 D GQDs, as exemplified by Shin et al. in a facile, novel sono-oxidation and photo-oxidation approach, using an acid-free neutral salt oxidant, oxone, which prevents the extra step of neutralization with a strong base; oxone is a low-cost and non-toxic oxidant. Graphene oxide sheets were used as the precursor, and uniform size distribution was obtained, between 2 and 6 nm [178]. This novel and versatile approach was followed by Nair et al., using KMnO_4_ as the oxidizing agent instead of oxone, delivering a good quantum yield (23.8%) and a product yield of >75%, with low cytotoxicity (tested up to 1000 μg/mL) and with half the time (30 min). Furthermore, it was applied successfully in bioimaging and sensing of Fe (III) ions [179]. During the same time, Shin et al. again came up with an acid-free one-pot solvothermal redox method for the synthesis of GQDs, using four different carbon precursors, namely graphite (G), MWCNTs, carbon fiber (CF), and charcoal (C). A strong blue UV-light illumination was exhibited by the synthesized G-GQDs, M-GQDs, CF-GQDs, and C-GQDs. Because of its simple purification and large-scale production, it could serve the application in industrial manufacturing with carbon precursors [180]. Lu et al. prepared GOQDs via a green and expeditious route, using carbon black as a precursor and hydrogen peroxide (H_2_O_2_) as the oxidant, following an acid-free one-pot hydrothermal method [181].

## 4. Comparison between Different Approaches for the Synthesis of GQDs

**Figure 15:**
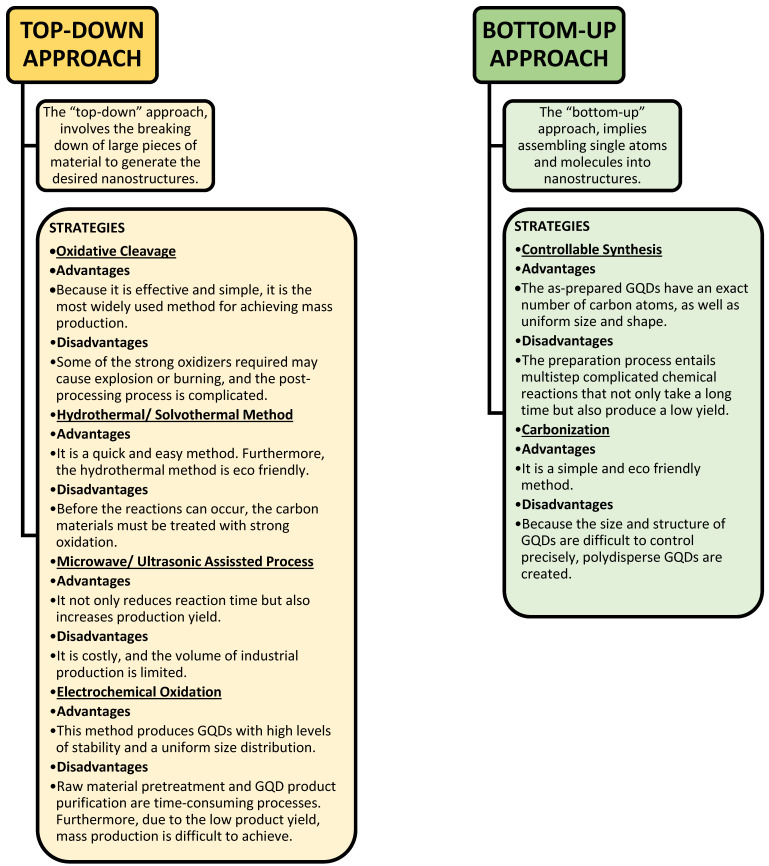
Advantages and disadvantages of various strategies of top-down and bottom-up approach for the greener synthesis of GQDs.

A systematic comparison between different top-down and bottom-up approaches is presented on the basis of the type of products generated, required capital, precursors used, and their applications; the relative advantages and disadvantages need to be balanced to make the appropriate selection for the desired application (Figure 15) [182].

## 5. Diverse Approaches for the Modification of GQDs

Various techniques have been explored to attain the definite and desired properties of GQDs, comprising modified and non-modified structures of GQDs, which can be further divided into three main categories:

1. Control over the size and shape;
2. Surface modification;
3. Heteroatom doping.

### 5.1. Size and Shape Control of GQDs

It is generally known that the photoluminescence phenomenon is more pronounced in GQDs due to quantum confinement and, in some way, is due to shape caused by edge effects [183,184]. To achieve observable control over the size and shape it is imperative to proceed through step-wise synthesis [185] as has been exemplified in the preparation of well-defined GQDs via the ruthenium-catalyzed cage opening of C_60_ molecules [186]. In another study, the growth of GQDs with the size of 2 to 6.5 nm was controllably achieved by causing a slight variation in the heating time from 3 to 10 min [149]. The shapes of GQDs can be varied from hexagonal, parallelogram, triangular, and trapezoid to mushroom-shaped by initiating a change in the annealing temperature and carbon clusters’ density [183].

**Figure 16:**
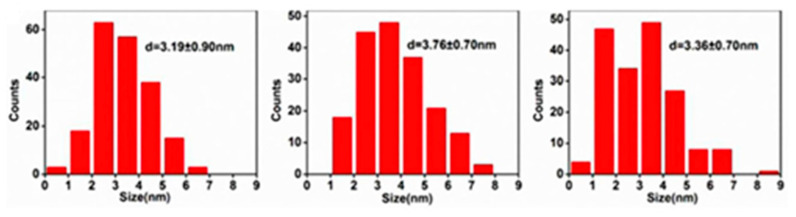
Size-distribution images for GQDs-PBS, GQDs-NaOH, and GQDs-KCl. Reprinted with permission from Ref. [187].

Figure 16 shows that the particle size of GQDs is roughly distributed between 1 and 9 nm. The statistical results indicate that the average particle diameters of GQDs-PBS, GQDs-NaOH, and GQDs-KCl are 3.19 ± 0.90, 3.76 ± 0.70, and 3.36 ± 0.70 nm, respectively. Furthermore, it has been reported that there is no significant difference in the shape or size of the samples, indicating that the three electrolytes have similar effects on the formation of GQDs [187].

### 5.2. Surface Modification of GQDs

**Figure 17:**
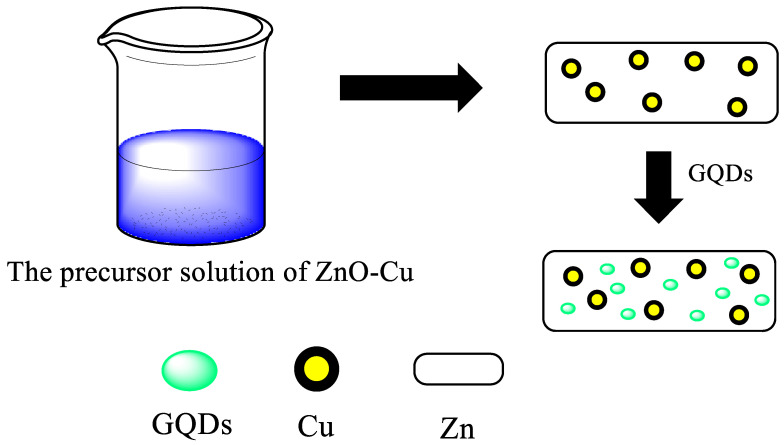
Schematic representation for the surface modification of GQDs.

Apart from dependency on shape and size, the PL properties of GQDs can be greatly tuned by using surface engineering, including surface oxidation [188], polymer passivation, and attachment of chemical species (Figure 17) [189], wherein the ZnO-Cu solution is used as a precursor and GQDs are employed onto the basal planes [190]. Introducing an oxygenated functional group on the basal plane of GQDs makes them hydrophilic, leading to ease of further functionalization. The quantum yield is known to be modified positively by the reduction of oxygenated GQDs, wherein emission is right-shifted in the case of the oxidation of GQDs. For example, the reduction of GQDs with NaBH_4_ blue-shifted the PL emission and increased the quantum yield by two [151,191,192]. Certain polymers have been reported for surface passivation, among which polyethylene glycol (PEG) is the most common; even with the addition of a thin layer of PEG, the quantum yield of GQD is doubled. However, this surface passivation has its limitations due to the complicated processes involved [31,193]. In a similar manner, the properties of biomass-waste-derived GQDs have been modified by surface engineering, particularly by the inclusion of certain chemical moieties and passivating agents. Suryawanshi et al. altered the GQDs derived from neem leaves via amine (–NH_2_) functionalization to increase the PL properties [33] when the green luminescence of the original GQDs was modified to blue after amine functionalization. This PL shift is attributed to the decrease in the agglomeration and changes in the oxygenated functional groups into –CONH_2_ and –C–NH_2_ surface groups, which suppress the non-radiative recombination path, resulting in the increase of PL intensity and quantum yield by a factor of two. Kalita et al. altered the GQDs with amine functionalization in a similar way to modify the quantum yield of rice-grain-derived GQDs [149]; the quantum yield was enhanced by 125% after amine functionalization because of the superior electron-donating tendency of amine groups. Wang et al. tuned GQDs obtained from coffee grounds with poly (ethylene imine) (PEI) to acquire the excitation-independent emission [148]. The blue fluorescence of the acquired GQDs changed to strong cyan fluorescence, and the quantum yield enhanced three-fold after PEI functionalization. The excitation-independent emission behavior of PEI-functionalized GQDs implies that the size and surface states of sp^2^ clusters contained in the GQDs should be more confined.

### 5.3. Heteroatom Doping of GQDs

Heteroatom doping has evolved lately as a novel technique for fine-tuning PL properties and the quantum yield of GQDs [194] where precursor materials are often exploited during synthesis; sulfur doping red-shifts the PL emission and increases the quantum yield. Wang et al. prepared a series of GQDs with controllable sulfur (S) doping using durian (biomass) as a precursor material [194] where the doping concentration was controlled through reaction time; doping falls linearly due to the removal effect of the heteroatom in sp^2^ carbon structure under longer reaction times. Moreover, it has been reported that N-doping [195] also offers a positive conversion of PL properties of GQD [193]. The catalytic properties and PL characteristics of GQDs have also been studied through doping with certain other elements, such as S [124], P [196], Si [197], and B [198]; for example, a high quantum yield of GQDs approaching 71% was obtained via S and N co-doping [198].

Table 1, Table 2, Table 3, Table 4, Table 5, Table 6, Table 7, Table 8 and Table 9 register the recent advances accomplished in the synthesis of GQDs during the past 10 years (2012–2021):

**Table 1:** The microwave-assisted methods for the preparation of GQDs.

Type of GQDs	Precursor	Size and Shape	Application	Reference
N-GQDs	Glucose	5.3 nm, spherical	Photo electronics and fluorescent probing	[102]
GQDs	*Clitoria ternatea*	10 nm, spherical	Treatment of Alzheimer’s disease	[103]
GQDs	Glucose	Less than 5 nm,	Detection of Al^3+^ ions	[199]
GQDs	Citric acid	Around 15 nm	For the synthesis of xanthenes	[200]
Graphene-TiO_2_ QDs	Graphene oxide	About 3 nm, spherical	In a photoelectrochemical cell	[201]
GQDs	Organic precursors	Spherical	Removal of triazine	[105]
N-GQDs	Citric acid and urea	Circular or elliptical	In optical, electronic, and biomedical devices	[106]
B-GQDs	Graphene oxide and borax	4 nm	Cell imaging	[107]
GQDs	Mangifera indica (mango)	2–8 nm	Near-infrared bioimaging and intracellular nano-thermometry	[108]
GQDs	Graphene oxide	-	ECL biosensing and imaging	[104]
B-GQDs	Bulk boron carbide (B_4_C) crystals	Around 6 nm	Dye-sensitized solar cell	[109]
His-GQDs	Citric acid and histidine	-	Supercapacitor	[202]
N-GQDs	Sodium citrate and triethanolamine	Around 5.6 nm	As fluorescent ink and in the detection of ferric ions	[112]
GQDs	Grape seed extract	50–60 nm	Cell Proliferation, Nucleus Imaging, and Photoluminescent Sensing	[113]
GQDs	Graphite	2–5 nm	-	[114]
GQDs	1,3,6-trinitropyrene	4.12 nm	Live-cell staining and white LEDs	[115]
GQDs	acetylacetone	5 nm, 2.3 nm	In enzyme-free biosensors, bioimaging and optoelectronic devices	[116]
Amine-functionalized GQDs	Glucose, H_2_O_2_, and NH_3_	3.78 nm, quasi-spherical	In visible-light photocatalytic systems	[203]
GQDs	aspartic acid and NH_4_HCO_3_ mixture	2.1 nm	Fluorescent probes for detection of iron ions and pH value	[204]
GQDS	De-oiled asphalt	2.65 nm,	As surfactant for asphalt emulsion	[205]
GQDs	Citric acid	-	Live-cell imaging	[117]
N-GQDs	Citric acid and semi-carbazide hydrochloride	4–8 nm,	Sensing and imaging	[206]
GQDs	Opuntia sp. extract	2.6 ± 0.63 nm, spherical	Phosphate detection	[207]
GQDs	Lemon juice	5–10 nm, spherical	Biomedical and chemical sensing	[208]

**Table 2:** The molecular carbonization methods for the preparation of GQDs.

Type of GQDs	Precursor	Size and Shape	Application	Reference
GQDs	Citric acid	Around 15 nm	-	[46]
GQDs	DI water and glucose	8 nm	-	[152]
GQDs	Citric acid	1.5 nm at pH 9, 1 nm at pH 10, below 2 nm at pH 12, spherical	-	[153]
GQDs	Corn powder	20–40 nm	Reduce charge recombination and increase free charge carriers	[161]
GQDs	Corn powder	20–40 nm	In dye-sensitized solar cells	[162]
GQDs	Polycyclic aromatic hydrocarbon	5–10 nm	Bioimaging and sensing of Fe^3+^ ions and hydrogen peroxide	[209]
N-GQDs	Citric acid	3.5 nm	Fe^3+^ ions detection	[163]
N-GQDs	Citric acid and urea	6 nm	Electrochemical sensing of hydroxyurea	[210]
Amino-functionalized GQDs	Asphalt	Less than 4 nm	Fe^3+^ detection	[168]
Nitrogen and phosphorus co-doped GQDs	Adenosine triphosphate	-	Cellular imaging	[164]
N-GQDs	Citric acid	5–10 nm, spherical	Cell labeling, bioimaging	[165]
GQDs	Citric acid and urea	5–10 nm, quasi-spherical	Optical, sensing, energy, and biological applications	[211]
GQDs	Glucose	Around 20 nm, spherical	Determination of free chlorine	[212]
N-GQDs	Orange juice	-	For turn-off sensing of TNP in an aqueous medium	[166]
N-GQDs	4-nitrophenol	5.2 nm	As metal-free photocatalysts for reduction of 4-nitrophenol	[167]
GQDs	En, Gl, Eg, Me, Dmf, Ac, Et, To, Ct	Less than 3 nm	-	[169]
GQDs	EDTA	Around 8.2 nm	Bioimaging and optoelectronic applications	[213]
GQDs	Honey	2.4 nm	As stable security ink and white-light emission	[150]
GQDs	Glucose	7–10 nm	As optical sensor for glucose	[214]
GQDs	L-cysteine	4–8 nm, elliptical	Selective sensing of curcumin	[215]
Amino-functionalized GQDs	Glucose	3–4 nm	Detection of copper ions	[216]
N-GQDs	Ammonium citrate	1–5 nm	Fabrication of BiOBr nanohybrids	[217]
N-GQDs	Ethanolamine	1–5 nm	Biological imaging, drug delivery	[218]
N-GQDs	Polyethylimine	7–12 nm	Heavy metal ions recognition and bio-labeling	[219]
GQDs	Citric acid	12.7 nm	Biomedical applications	[220]
GQDs	Glucose and citric acid	22 nm, 8 nm	As fluorescence probe for the detection of Au(III) ion	[221]
N-GQDs	Blue citric acid and glycine	3–8 nm	Nanomedicine	[222]

**Table 3:** The soft template methods for the preparation of GQDs.

Type of GQDs	Precursor	Size and Shape	Application	Reference
S-GQDs	Paraffin oil and carbon disulfide	2.46 nm, spherical	High-performance optoelectronic devices	[174]
N-GQDs	Citric acid	-	Phosphor-based light-emitting diodes	[175]
Histidine-functionalized GQDs	Citric acid and histidine	3.2 nm	For the synthesis of polystyrene microspheres for adsorption of Cu^2+^	[176]
Histidine-functionalized GQDs	Citric acid and histidine	-	Volumetric sensing of dopamine	[177]
GQDs	HBC	60 nm, disk-like	-	[173]

**Table 4:** The hydrothermal and microwave-assisted hydrothermal methods for the preparation of GQDs.

Type of GQDs	Precursor	Size and Shape	Application	Reference
GQDs	Coffee grounds	-	Sensing of heavy metals	[84]
GQDs	H_2_O_2_	35 nm	Cellular imaging, drug delivery	[85]
GQDs	Graphene sheets	10 nm (diameter)	Biological labeling	[16]
GQDs	Glucose	1.65 nm, spherical	DUV photonic devices	[120]
GQDs	Glucose	<5 nm ± 0.55, spherical	Optical applications	[121]
p-GQDs	Graphene Oxide	2–6 nm	Biological optoelectronic	[122]
GQDs-F, GQDs-P	Fluorinated graphene oxide	GQDs-F- 5.6 nm GQDs-P- 6.0 nm	Solar cells, biosensors, and bioimaging	[123]
GQDs	Citric acid	10 nm	Adsorbent for toxic carbamate pesticide oxamyl	[124]
N-GQDs	Urea	3 nm, crystalline structure	Biolabeling and bioimaging	[125]
N-GQDs	Glucose	3–4 nm, spherical	Dopamine sensors	[126]
WGQDs	Graphite	Lateral size 1.25–2.75 nm	WLEDs	[127]
FGQDs	Glucose	<3 nm	Biomedical applications, treatment of amyloidosis	[223]
GODQs	Graphene oxide	7–12 nm, zigzag structure	biosensing, imaging, and labeling	[224]
GQDs	Glucose	1.1 nm, spherical	Supercapacitors	[225]
RGQDs and NGQDs	Graphene oxide and glucosamine precursors	3–6 nm size	Imaging-based Temperature sensors	[226]

**Table 5:** The biomass-derived methods for the preparation of GQDs.

Type of GQDs	Precursor	Size and Shape	Application	Reference
a-GQDs, b-GQDs, c-GQDs	Anthracite, bituminous, coke	b-GQDs- 2.96 ± 0.96 nm, crystalline hexagonal c-GQDs- 5.8 ± 1.7 nm a-GQDs- 29 ± 11 nm	Bioimaging, biomedicine, photovoltaics, and optoelectronics	[59]
GQDs	low-cost coal	20 ± 10.25 nm,	Bioimaging applications	[133]
Neem-derived GQDs, fenugreek-derived GQDs	Neem and Fenugreek leaves	Neem- 2–8 nm, spherical Fenugreek- 3–10 nm, spherical	White LEDs	[31]
Am-GQDs	Neem leaves	5–6 nm, crystalline	Soil and water diagnosis, food safety surveillance	[33]
N-GQDs	Marigold	3.2 nm, uniform crystal lattice	Detection of Fe^3+^ in water, bioimaging applications	[134]
GODQs	Paper waste	1.2 nm, spherical	Optical	[136]
GQDs	CTP	1.7 ± 0.4 nm, hexagonal lattice	Applications in aqueous systems	[137]
GQDs	Graphitic waste	10 ± 0.5 nm, spherical	Humidity sensors	[139]
GQDs	Sugarcane bagasse	2–6 nm, hexagonal	Nanoprobes for multicolor bioimaging	[140]
GQDs	Cellulose polymer	1–5 nm	Bioimaging and biolabeling	[69]
GQDs	Spent tea	1.6 ± 0.55 nm, uneven shape (GQDs-500)	Biomolecules and metal ion sensors	[227]
RH-GQDs	Rice husk	3–6 nm, crystalline lattice	Fluorescent bio-probes	[32]
LGQDs	Alkali lignin	-	Ultrasensitive biosensors	[228]
N-S co-doped GQDs	Graphite waste (powdered)	10 ± 0.5 nm, spherical	Humidity sensing	[138]
Reduced-GQDs (rGQDs)	Glucose	nearly spherical, 4–14 nm	Electrochemical applications	[229]
NIR-GQDs (Near infrared GQDs)	*cis*-cyclobutane-1,2-dicarboxylic acid (CBDA-2)	6.5 ± 3.5 nm	Cell imaging and metal ion detection	[230]
N-GQDs	Arjuna bark, waste melamine sponge	2–3 nm, spherical	Bioimaging, H_2_O_2_ sensing	[141]
N-GQDs	Pineapple leaf fiber	3.7 nm, spherical	Hg^2+^ detection	[231]
GQDs	Marigold flower (*Tagetes erecta*)	~5.7 nm	Supercapacitors	[135]
GQDs	Corn straw	2.67 nm	PO_4_^3−^ detection	[232]

**Table 6:** The ultrasonic facilitated methods for the preparation of GQDs.

Type of GQDs	Precursor	Size and Shape	Application	Reference
GQDs	-	3–5 nm	Biosensors, bioimaging, laser, and light-emitting diodes	[70]
GQDs	Graphene oxide	-	Sensing of alkaline phosphatase	[71]
GQDs	Ethyl acetoacetate, NaOH	2–10 nm, spherical	Biophotonic	[72]
GQDs	Graphene oxide	4–10 nm	Solar cells, fluorescent detection, cellular imaging	[73]
GQDs	Leather	5 nm, spherical	High-contrast bioimaging, biosensing, photovoltaics, and drug delivery	[74]
GQDs	Graphene flakes	2–4 nm	Thin-film electronic and optoelectronic devices	[75]
PGQDs, EGQDs, GOGQDs	Natural graphite, expanded graphite, and oxide graphite	2~4 nm	-	[76]
LD-GQDs, HD-GQDs	Graphitic carbon	HD-GQDs- 2–6 nm, curved shape and zigzag edges LD-GQDs- 2–9 nm, rectangle, and hexagon	Biology, electronic, energy, and engineering	[77]
GQDs	Graphene oxide	5–7 nm	Optoelectronic applications	[78]
C-GQDs	Coal	3.2 ± 1.0 nm,	Sensor applications	[79]
GQDs	Miscanthus	-	-	[233]

**Table 7:** The oxidative cleavage methods for the preparation of GQDs.

Type of GQD	Precursor	Size and Shape	Application	Reference
GQDs	MWCNT	Less than 5 nm, zigzag structure	-	[99]
GQDs	Carbon fibers	1–4 nm	Bioimaging and biosensing	[18]
GQDs	Graphene oxide	-	Bioscience and energy applications	[193]
GQDs	Graphene nanoribbons	100–200 nm	Humidity/pressure sensing applications	[234]
GQDs	Carbon black	Less than 10 nm	DSSCs	[100]
GQDs	Graphene oxide	1–3 nm	-	[94]
GQDs	GO	15 nm, Spherical	Sensing	[235]
N-GQDs	Low-cost graphite	5 nm	Bioimaging	[86]
GQDs	GO	20–30 nm, quasi-hexagonal	Drug-delivery applications	[236]
Carbon fiber GQDs (CF-GQDs), Ethiopia GQDs (E-GQDs), Mandheling GQDs (M-GQDs), and Kenya AA GQDs (K-GQDs)	Coal-like precursors: carbon fiber, Ethiopia, Mandheling, and Kenya AA	2.60 ± 0.58 nm, 3.02 ± 0.913 nm, 3.66 ± 0.84 nm, and 3.69 ± 0.98 nm	Biomedicine	[237]

**Table 8:** The oxidation (free-radical, chemical, etc.) methods for the preparation of GQDs.

Type of GQD	Precursor	Size and Shape	Application	Reference
Porous graphene (PGN)	Graphene oxide	5–150 nm	Detectable molecular separation	[65]
GQDs	Graphene oxide, H_2_O_2_	1–6 nm	-	[66]
Graphene nanosheets	Carbon nanotubes	1–3 nm	Supercapacitors, solar cells, etc.	[81]
GQDs	Vulcan XC-72 carbon black	-	-	[83]
GQDs	C_60_ cage	-	Effective peroxidase mimic	[82]
CQDs	Chinese ink	1–6 nm	Electrochemical and photoluminescent applications	[238]

**Table 9:** The acid-free methods for the preparation of GQDs.

Type of GQD	Precursor	Size and Shape	Application	Reference
GQDs	Graphene oxide	4–5 nm, crystalline	-	[178]
GQDs	Graphene oxide	1–5 nm, hexagonal lattice	Cancer detection or bioimaging	[179]
GQDs	Graphite (G), MWCNTs, carbon fiber (CF), and charcoal (C)	2–8 nm, crystalline lattice	Industrial manufacturing with carbon precursors	[180]
Graphene oxide quantum dots	Black carbon	3–4.5 nm	Bioimaging and biological applications	[181]

## 6. Characterization Techniques and Structural Analysis of GQDs

The characterization of the GQDs follows the traditional processes, such as nuclear magnetic resonance (NMR), transmission electron microscope (TEM), X-ray diffraction (XRD), Fourier-transform infrared spectroscopy (FTIR), photo-luminescence, UV spectroscopy, and Raman spectroscopy [32,75].

### 6.1. Fourier-Transform Infrared Spectroscopy (FTIR)

Studies pertaining to the use of FTIR revealed that GQDs and CQDs usually contained oxygen, hydrogen, and carbon; moreover, FTIR is a robust device to analyze the oxygen-containing groups on basal planes of GQDs. As an example, *N*-doped GQDs were synthesized by a hydrothermal method via the pyrolysis of citric acid as the carbon source and urea as the nitrogen source, and the presence of doped nitrogen in GQDs and methotrexate -(N-GQDs) was confirmed by FTIR characterization [239].

In addition, it is possible to utilize infrared spectroscopy for modified carbon-based QDs to determine the effective passivation. Iannazzo et al. prepared the GQDs having a diameter in the range of 5 nm via acidic oxidation of pristine multiwalled carbon nanotubes (MWCNTs). The intense wide peaks were obtained at ~1620 cm^−1^ and 3450 cm^−1^, which were related to O–H vibrations and C=O bonds, respectively; the peak at 1072 cm^−1^ was matched up with the C–O alkoxy groups. Thus, the presence of various oxygenated functional groups on the surface of the GQD could be discerned; they are responsible for their high solubility in water [240].

### 6.2. Transmission Electron Microscopy (TEM)

Besides other techniques used for nanomaterial, TEM [241] has been extensively deployed for determining the shapes, sizes, and dispersion of GQDs, and with a high resolution of 0.1–0.2 nm, it could identify the ultra-structure of the samples. Jing et al. prepared CQDs by using biomass as a precursor via the hydrothermal technique when TEM revealed their microstructure to have a uniform diameter in the range between 1.5 and 4.0 nm. Notably, the high-resolution TEM image reveals lattice fringes with 0.20 nm of inter-planar space that is corresponding to the facet of graphitic carbon [242].

### 6.3. UV Spectroscopy

GQDs have numerous applications in photon harvesting in the short wavelength area due to the p–p * transition of the C double bonds, and, hence, these dots exhibit stronger optical adsorption in the UV zone (260 to 320 nm). GQDs demonstrate a sharp peak ranging between 270 and 320 nm that shows the probable contribution to the n–p* transition of the CO double bond [30]. In another study, edge-controlled and strongly fluorescent GQDs with the layered structure were procured by using diverse solvents. An elegant PL emission of the GQDs was obtained in distinct liquid solutions; a high QY of 32% for PL was observed for prepared GQDs in DMF solvent [243]. The UV–Vis absorption spectrum of N-GQDs revealed a broad absorption shoulder, ~255 nm, which originated due to π → π * of C=C bonds; N-GQDs aqueous solution exhibited a bright blue emission when exposed to UV light that was further validated by fluorescence analysis, which displayed the emission wavelength at 443 nm when excited at 340 nm [239].

### 6.4. Other Spectroscopy Studies

Nuclear magnetic resonance (NMR) can be deployed for analyzing the hybrid kinds of the carbon atoms in the crystalline lattices and the type of interaction between the carbon atoms, thus providing further structural insights on carbon-based QDs. As an example, the ^1^HNMR spectrum of GQDs, via pyrolysis of citric acid in the presence of octylamine on the catalyst, revealed appropriate peaks for the modified graphene. There is a sharp peak close to 1.25 ppm and a peak of about 7.5 ppm corresponding to the H atom, which is connected to the amine groups and bonds between the octyl of a carbon group on the GQDs. ^13^C-NMR spectroscopy could differentiate sp^2^- and sp^3^-hybridized carbons and reveal the carboxyl and amide groups signals ranging between 170 and 185 ppm; ^13^C-NMR confirmed the connection of alkyl groups to the graphene surface [244,245].

Thambiraj et al. reported a greener method to prepare fluorescent CQDs by using the sugarcane bagasse and exfoliation, as well as the chemical oxidation. XRD characteristic peaks have been observed at 2Ɵ = 11.4 °C, 20.6 °C, 22.8 °C, 42.3 °C, and 45.7 °C. These diffraction pattern studies indicate the face-centered cubic crystal structure of CQDs [246].

Raman spectroscopy is a spectroscopic technique endowed with a high resolution for the characterization of the lattice structure and the electronic and optical properties of carbon materials [247]. The Raman spectra of GQDs indicated the presence of D and G bands, as well as higher-order modes (2D, D + G, and 2G). The D- and G-band frequencies and intensity were found to increase with the increase in the size of GQD, while higher-order modes (2D, D + G, and 2G) were also enhanced in intensity and became more well-defined. Usually, Raman spectroscopy is a good diagnostic tool for evaluating the formation of bottom-up synthesized GQDs [248].

## 7. Concluding Remarks and Future Outlook

Graphene quantum dots (GQDs) are widely regarded as a present-generation carbon material owing to their exceptional optical and electrical properties that are suitable for many technological and energy production fields [34,35,36]; insight into recent advances in the greener assembly of GQDs is presented in this overview. As has been examined in the literature, the size of GQDs has an inordinate influence on their operation in different energy appliances; nonetheless, a systematic study that is inclusive of their size is still needed in the future. Although research on GQDs has moved from its state of infancy, advanced techniques for their synthesis are required to attain precise control over the property–application relationships, especially those that are friendly to the environment. Nonetheless, this aim has to be well-balanced in terms of associated costs and large-scale production. Lingering problems, namely the lack of conclusive evidence for the PL mechanism of GQDs, are yet to be settled and require further detailed investigations. The necessary gap between the synthesis and characterization of GQDs shall be filled to accelerate and realize the great potential of novel GQDs. The bottom-up approach is a relatively more explored method over the top-down tactic according to available research data and the conclusions attained from such studies; delineation of the simpler underlying mechanisms and exactly transcribing the diminutive differences among the oxidation methods are still warranted. Despite the fact that GQDs are often prepared by using biomass, not all methods resulted in a high yield of GQDs, and this is, indeed, a drawback that needs to be attended to in the near future. Notably, most of the biomass-derived GQDs have not been represented in the electrochemical sensing appliances with high sensitivity, a void that needs to be filled among the advanced electrochemical devices. Certainly, there is a tremendous future scope in exploring newer “greener” precursors and preferably agricultural residues or wastes that can be deployed for the synthesis of GQDs, which are least burdensome on the environment, thus embracing the sustainability theme [249].

The immediate goal in this emerging field ought to find photochemical solutions for increasingly ambitious synthetic goals. Long-term goals should include enhanced efficiency and synthetic utility, as well as eventually accomplishing chemical synthesis under sunlight. The combination of C-dots doping and C-dots-based nanocomposites with other nanomaterials may open up new avenues for systematically studying the effect of structural parameters and chemical compositions on electrocatalyst catalytic performance, leading to fundamental insights and practical applications. This review will, hopefully, stimulate further research in the investigation of potential eco-friendly precursors and greener synthetic routes for the production of GQDs on a larger scale, and the required advances obligatory in this field will be met in the near future by the joint efforts of multidisciplinary research teams.

Carbon dots will undoubtedly improve people’s lives in the future by paving the way for simplified bioimaging techniques, efficient optoelectronics, drug delivery systems, optical sensors, photovoltaic systems, and detectors. Because this is a relatively emerging field, there is plenty of room for new discoveries and further investigations. We believe that new physical and chemical properties will be discovered, as well as new applications and devices. Although many challenges may be encountered, they may also lead to many more benefits that will help achieve a better future.

## Data Availability

Not applicable.

## Abbreviations

GQDs	graphene quantum dots
NPs	nanoparticles
CNTs	carbon nanotubes
GO	graphene oxide
N-GQDs	nitrogen-doped graphene quantum dots
PGQDs	pristine graphene quantum dots
EGQDs	expanded graphene quantum dots
GOGQDs	graphene oxide graphene quantum dots
LD-GQDs	low-defects graphene quantum dots
HD-GQDs	high-defects graphene quantum dots
C-GQDs	coal-derived graphene quantum dots
F-GQDs	fenugreek-derived graphene quantum dots
CQDs	carbon quantum dots
PL	photoluminescence
TATB	1,3,5-triamino-2,4,6-trinitrobenzene
HBC	hexa-peri-hexabenzocoronene
CA	citric acid
His-GQD-GMA	histidine–graphene quantum dot–graphene micro-aerogel
ATP	adenosine triphosphate
N and P co-doped GQDs	graphene quantum dots co-doped with nitrogen and phosphorus
QY	quantum yield
FL	fluorescence
TNP	2,4,6-trinitrophenol
En	ethylenediamine
Gl	glycerol
Eg	ethylene glycol
Me	methanol
Dmf	dimethyl formative
Ac	acetone
Et	ethanol
To	toluene
Ct	carbon tetrachloride
PEI	polyethyleneimine
N-GO	nitrogen-doped graphene oxide
MWCNT	multiwalled carbon nanotube
G-GQDs	graphite-derived graphene quantum dots
M-GQDs	MWCNT-derived graphene quantum dots
CF-GQDs	carbon fiber-derived graphene quantum dots
C-GQDs	charcoal-derived graphene quantum dots
a-GQDs	anthracite-derived GQDs
b-GQDs	bituminous coal-derived GQDs
c-GQDs	coke-derived GQDs
PLAL	pulsed laser ablation in liquid
GOQDs	graphene oxide quantum dots
mGQDs	*Mangifera indica* derived GQDs
RH-GQDs	rice-husk-derived GQDs
LGQDs	lignin-derived GQDs
MAH	microwave-assisted hydrothermal
WGQDs	white fluorescent GQDs
WLEDs	white-light-emitting diodes
CL	chemiluminescence
DSSC	dye-sensitized solar cells
XRD	X-ray diffraction
TEM	transmission electron microscope
FITR	Fourier-transform infrared spectroscopy
ETL	electron transport layer
